# Gene-Focused Networks Underlying Phenotypic Convergence in a Systematically Phenotyped Cohort With Heterogeneous Intellectual Disability

**DOI:** 10.3389/fbioe.2020.00045

**Published:** 2020-02-07

**Authors:** Yan Wang, Li-Na Zhu, Xiu-Wei Ma, Fang Yang, Xi-Lin Xu, Yao Yang, Xiao Yang, Wei Peng, Wan-Qiao Zhang, Jin-Yu Liang, Wei-Dong Zhu, Tai-Jiao Jiang, Xin-Lei Zhang, Zhi-Chun Feng

**Affiliations:** ^1^BaYi Children’s Hospital, The Seventh Medical Center of PLA General Hospital, Beijing, China; ^2^National Engineering Laboratory for Birth Defects Prevention and Control of Key Technology, Beijing, China; ^3^Beijing Key Laboratory of Pediatric Organ Failure, Beijing, China; ^4^Suzhou Institute of Systems Medicine, Chinese Academy of Medical Sciences, Suzhou, China; ^5^The Second People’s Hospital of Aohanqi, Inner Mongolia, China; ^6^Center of Systems Medicine, Institute of Basic Medical Sciences, Chinese Academy of Medical Sciences & Peking Union Medical College, Beijing, China; ^7^Suzhou Geneworks Technology Co., Ltd., Suzhou, China

**Keywords:** intellectual disability, phenotypic convergence, gene-focused networks, co-occurrent phenotype, machine learning, pathogenic genes prediction

## Abstract

The broad spectrum of intellectual disability (ID) patients’ clinical manifestations, the heterogeneity of ID genetic variation, and the diversity of the phenotypic variation represent major challenges for ID diagnosis. By exploiting a manually curated systematic phenotyping cohort of 3803 patients harboring ID, we identified 704 pathogenic genes, 3848 pathogenic sites, and 2075 standard phenotypes for underlying molecular perturbations and their phenotypic impact. We found the positive correlation between the number of phenotypes and that of patients that revealed their extreme heterogeneities, and the relative contribution of multiple determinants to the heterogeneity of ID phenotypes. Nevertheless, despite the extreme heterogeneity in phenotypes, the ID genes had a specific bias of mutation types, and the top 44 genes that ranked by the number of patients accounted for 39.9% of total patients. More interesting, enriched co-occurrent phenotypes and co-occurrent phenotype networks for each gene had the potential for prioritizing ID genes, further exhibited the convergences of ID phenotypes. Then we established a predictor called IDpred using machine learning methods for ID pathogenic genes prediction. Using10-fold cross-validation, our evaluation shows remarkable AUC values for IDpred (auc = 0.978), demonstrating the robustness and reliability of our tool. Besides, we built the most comprehensive database of ID phenotyped cohort to date: IDminer http://218.4.234.74:3100/IDminer/, which included the curated ID data and integrated IDpred tool for both clinical and experimental researchers. The IDminer serves as an important resource and user-friendly interface to help researchers investigate ID data, and provide important implications for the diagnosis and pathogenesis of developmental disorders of cognition.

## Introduction

Intellectual disability (ID), also known as mental retardation, is characterized by significant impairment in cognition. The patients with ID usually have the obvious shortcomings of adaptive behavior before the age of 18, and a high incidence rate, 1–3%, making it a worldwide social problem (Maulik et al., 2011; Mefford et al., 2012). It can occur in isolation or in combination with congenital malformations or other neurological features such as epilepsy, congenital malformations, sensory impairment, and autism spectrum disorders (ASD), and its severity (mild, moderate, severe, and profound) is highly variable (Vissers et al., 2016). The heterogeneity of phenotypes poses additional challenges for understanding the complex etiology, with contributions by environmental factors, perinatal hypoxia, and genetic factors. In recent years, genetic factors including chromosomal abnormalities, single and multiple gene mutations have found to become increasingly prominent for the disease (Gilissen et al., 2014; Lelieveld et al., 2016; Reichenberg et al., 2016). With the increasing number of ID cases identified in clinics, its phenotypes have found to be extremely heterogeneous. Previous studies found that patients with identical mutations in a single gene could give rise to different phenotypes (Hoischen et al., 2014). As the limitations of detection technologies and the heterogeneity of ID genes and phenotypes, many patients still lack appropriate diagnosis.

In the past 10 years, a large number of studies have been carried out in order to explore the genetic mechanism of ID (Gécz et al., 2009; Ellison et al., 2013). In particular, the development of second-generation sequencing technology facilitates the rapid investigation of more DNA samples from ID cases (Rauch et al., 2012; Gilissen et al., 2014). This led to an expansion in the number of genes associated with ID. Having mass data about ID genes, clinical phenotypes, and pedigrees available in the public domain could shed insights into ID mechanisms. A previous report suggests that ID genes are substantially enriched with co-expression, protein-protein interactions, and specific biological functions. Furthermore, they also revealed combinations of typical phenotypes within process-defined groups of ID disorders by clusters of ID genes with significantly elevated biological coherence (Kochinke et al., 2016). This suggests that ID genes and phenotypes have their own characteristics, and these data can be used to define mechanisms of ID and may improve the diagnosis of patients.

In this study, the ID genes, phenotypes, and pedigrees were extracted manually and analyzed and then integrated to build a standard ID database IDminer, which analyzed the phenotypes, genes, families and their relationships based on the individual patient. Furthermore, the candidate pathogenic genes for ID patients could be prioritized based on the molecular feature of ID genes and the genes specific phenotypes and phenotypic pairs. Furthermore, the similarity between patients was also evaluated via clinical features and could help patients with effective intervention. Importantly, the curated data including ID phenotypes, genes and pedigrees, their integrated analysis and their applications are accessible online via http://218.4.234.74:3100/IDminer/.

## Materials and Methods

### Analysis of Specific Phenotypes and Phenotypic Pairs

Each pathogenic gene could be associated with multiple patient samples, and each patient may have different phenotypes. For each gene, the specific phenotypes were obtained with the enrichment analysis using the hypergeometric distribution. A gene could correspond to multiple patients. For each patient, any two of their phenotypes formed a phenotype pair, referred to as co-occurrence. A phenotype pair could appear in *N* patients (*N* represents the frequency of phenotypic pairs). In situations with a single gene affecting multiple cases, multi-phenotypic pairs and their frequencies were obtained. For each phenotypic pair, we analyzed whether the co-occurrence was enriched in the affected patients or not.

### Construction of Co-occurrence Network

For constructing a co-occurrence network, all phenotypic pairs with a *P*-value = 0.05 for at least one gene were built as a non-directional network. In this network, each node represents a phenotype and the node size indicates the frequency of the phenotype in the database, while the edges denote significantly enrichment between phenotype pairs. Then the modules were extracted with the R igraph package.

### Phenotype-Based Samples Similarity Analysis

The same phenotype may appear in different patient samples. Based on the number of the same phenotypes between these samples, similarity scores between pairs of patient samples was calculated.

### The Phenotype Converting Tool

The tool was used to calculate the similarity between the users’ input phenotypes and the 2,075 standard phenotypes in this website. The python module named FuzzyWuzzy was used to calculate the similarity score [0,100]. The higher the score, the more similar the two phenotypes.

### Supervised Machine Learning Prediction

In this study, the supervised machine learning method, Support Vector Machine (SVM), was employed for ID pathogenic genes prediction. The R language interface of LIBSVM was used to construct the SVM-based pathogenic predictors. The radial basis function was chosen as the kernel function, and the other parameters were set at the default. A prediction model was trained using repeated 10-fold cross-validation of the training dataset, and their predictive performance was evaluated in the independent test dataset.

### Web Interface Configuration

The interface has two main parts: one part displayed the ID knowledge base data and the search results, while the other displayed the input and results of the analysis tool. Through the search box on the main page, users could search for a gene or a phenotype. Through the tools button in the main menu, users could enter the analysis interface, and according to the given phenotypes and genes, the ID genes were identified, and the association between the genes and their phenotypes were visualized. The web service was mainly based on java server pages, JavaScript, R, Python, Ajax, Apache, and MySQL.

## Results

### Data Curation

**FIGURE 1:**
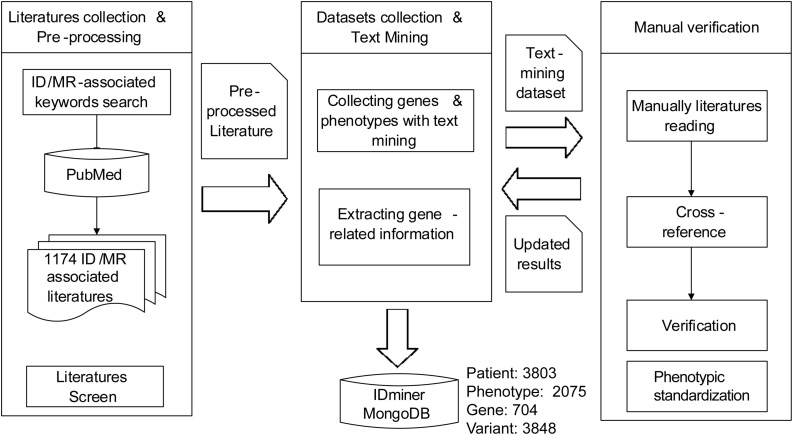
The flowchart of data collection and curation. The framework for genes extracting, paper downloading, phenotypes, and pedigrees obtaining and data curating of this project.

We first employed the keywords, such as ID, mental retardation, developmental delay, cognitive impairment, developmental disability, and learning disability to accomplish the literature searches by using PubMed. Then the literature was filtered through the artificial proofing method, and the ID-related papers and genes were retained. The text mining method was used to mark phenotypes in the literature using the HPO^1^ database phenotypic information as a reference. Then the gene name, mutation site, and phenotypes were curated manually (Figure 1). Based on the sample description in the literature, the family information of the samples were also collated from the HGNC (HUGO Gene Nomenclature Committee) database according to the acquired ID-related gene name information, such as gene alias, chromosome localization, corresponding OMIM ID, and Ensembl ID, and the biological function and pathway information for these genes were marked simultaneously through GO^2^ and KEGG^3^ databases.

### The Landscape and Convergence of ID Genes

**FIGURE 2:**
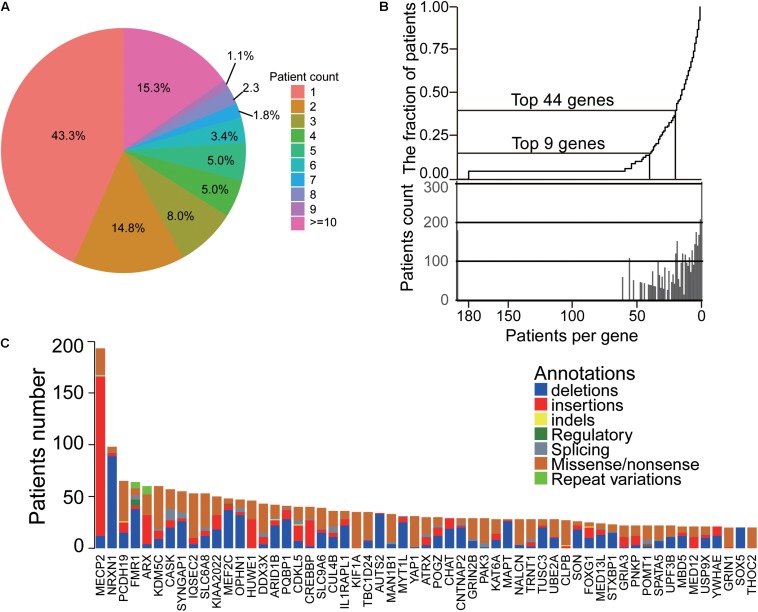
The landscape and convergence of ID genes. **(A)** The distribution of patient number for each gene. Most genes had less than three patients. **(B)** The top genes accounted for most patients. **(C)** The heatmap of genes and their mutations/indels in ID patients.

Through 1174 ID papers, we obtained a total of 3803 samples with 2075 phenotypic descriptions, that were caused by 704 ID genes. Among these genes, there are 3848 mutations, containing 1793 missense/non-sense mutations, 182 splicings, and 610 indels. We found that the majority of the genes were identified in less than 10 patients, and 305 genes (43.3%) found in only one patient and 103 genes (14.8%) in two patients (Figure 2A). Also, a small set of genes caused more patients than other genes, as shown in Figure 2B, the top nine genes ranked by the number of patients accounted for 14.9% of the total patient group, and the top 44 genes included 39.9% of patients. Moreover, our analysis also showed some ID genes had the dominant mutation types (Figure 2C). For the top 57 genes ranked by the number of ID patients, the majority of mutations of patients harboring mutated MECP2, HUWE1, and CREBBP are gross insertions. In addition, the predominant mutation type of patients with mutated THOC2, KIF1A, KDM5C, IQSEC2, SLC6A8, TBC1D24, MAN1B1, YAP1, GRIN2B, PAK3, NALCN, CLPB, and GRIN1 genes are missense/non-sense mutations, while deletions are mainly found in patients harboring SOX4, NRXN1, FMR1, MEF2C, OPHN1, PQBP1, AUTS1, MYT1L, CNTNAP2, MAPT, and TUSC3 genes. Importantly, the mutation types of 47 of the top 57 genes contained gross insertions (most duplications) and missense/non-sense, suggesting that both deletion and overexpression of these genes were likely to cause ID disease. These findings suggested that despite the diversity of ID genetic variation, most ID patients are caused by a small number of genes based on its genetic bias and convergence.

### The Heterogeneities of ID Phenotypes

**FIGURE 3:**
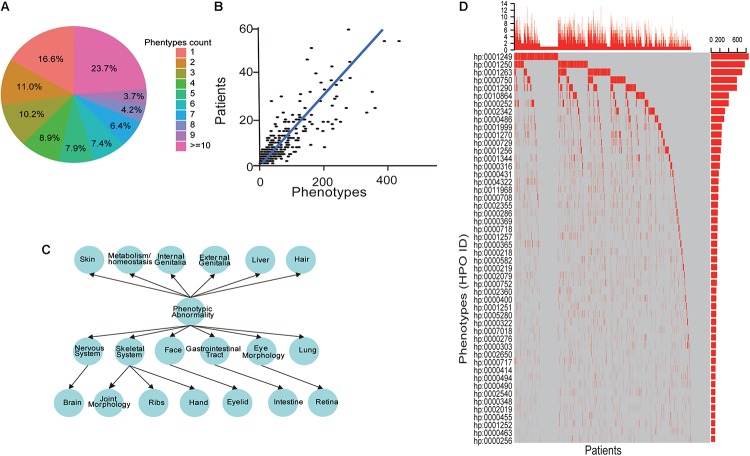
The heterogeneities of ID phenotypes. **(A)** The distribution of the phenotypes number for each patient. **(B)** A scatter point and line fitting showing the correlation between the patient number and phenotypes count. The patient number and phenotype count were derived from each gene in the database. **(C)** The phenotypes structure of ID patients. **(D)** The oncoprint-like representation of phenotypes in ID patients.

Among the patient cohort, 637 (16.6%) patients have a unique phenotype, while 901 (23.7%) patients have more than ten phenotypes (Figure 3A). Also, our data showed that the number of phenotypes for each patient had a positive correlation with the number of the patients, which showed a significant linear relationship (Spearman *P*-value < 0.001, Figure 3B) and indicated the heterogeneity of the ID phenotypes. Additionally, HPO structure analysis found the accompanying phenotypes of ID were also widely distributed, including symptoms in many parts of the body (Figure 3C). For these phenotypes, as shown in Figure 3D, the top 50 phenotypes ranked by the number of patients exhibited that the ID was usually accompanied by other mental diseases, such as seizure, epilepsy, microcephaly, ataxia, microcephaly and autism, abnormal behaviors containing hypotonia, strabismus, sleep disturbance, constipation, delayed or absent speech, motor delay, hyperactivity, feeding difficulties and inability to walk, and dysmorphism about spine, face, stature, and cryptorchidism. These results showed that the phenotypes of ID patients had extreme heterogeneity.

### The Convergences of ID Phenotypes

**FIGURE 4:**
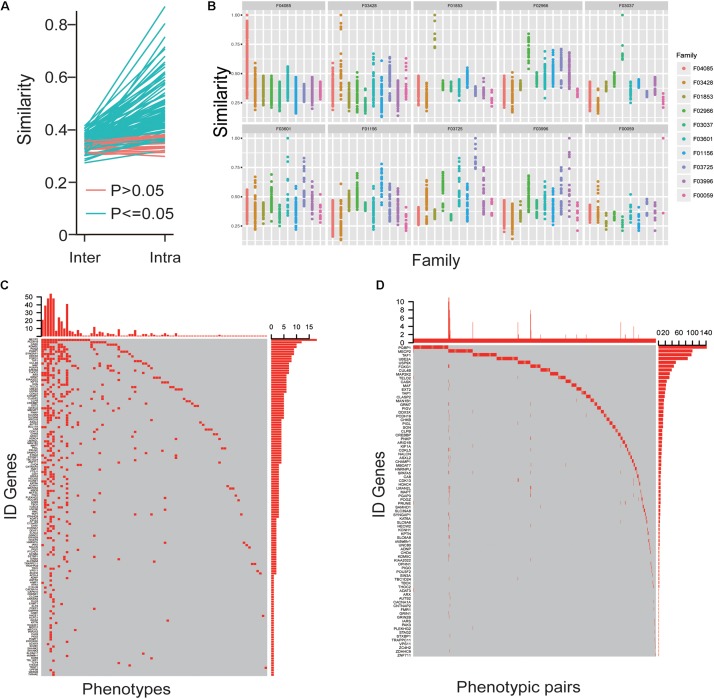
The convergences of ID phenotypes. **(A)** The mean value of intra-similarity between patients caused by one gene was higher than the mean value of inter-similarity between the gene’s patients and other genes’ patients. **(B)** The similarity of phenotypes in patients caused by identical mutations among the same and other families. **(C)** The oncoprint-like representation of specific phenotypes for genes. **(D)** The oncoprint-like representation of specific phenotypic pairs for genes.

The phenotypes that were converged for each gene based on the fact that intra-similarity between patients caused by one gene were more than inter-similarity between different genes’ patients (Figure 4A) and the phenotypes in patients caused by the identified mutations in the same family had more similarity than other families (Figure 4B). To better understand the convergence of the ID clinic features, we first obtained the specific phenotypes for each ID gene with enrichment analysis. A total of 143 phenotypes, appearing in at least five patients caused by the same gene, were enriched in some genes’ patients (Figure 4C). Importantly, among the phenotypes, 47 appeared in only single gene’s patients and accounted for 30 genes, which could help to diagnosis the patients caused by the genes (Figure 4C). To illustrate the relationships between phenotypes, we also investigated the situation of two phenotypes could be co-occurred in one patient, and the co-occurrence phenotypes were recorded as “phenotypic pairs.” We analyzed these phenotypic pairs presented in patients with an enrichment analysis. Interestingly, we found that most enriched phenotypic pairs were specific for a single gene. Like single phenotype analysis, phenotypic pairs made it easy to diagnosis patients with 82 ID genes (Figure 4D).

### Gene-Focused Network for Phenotype Enrichment

**FIGURE 5:**
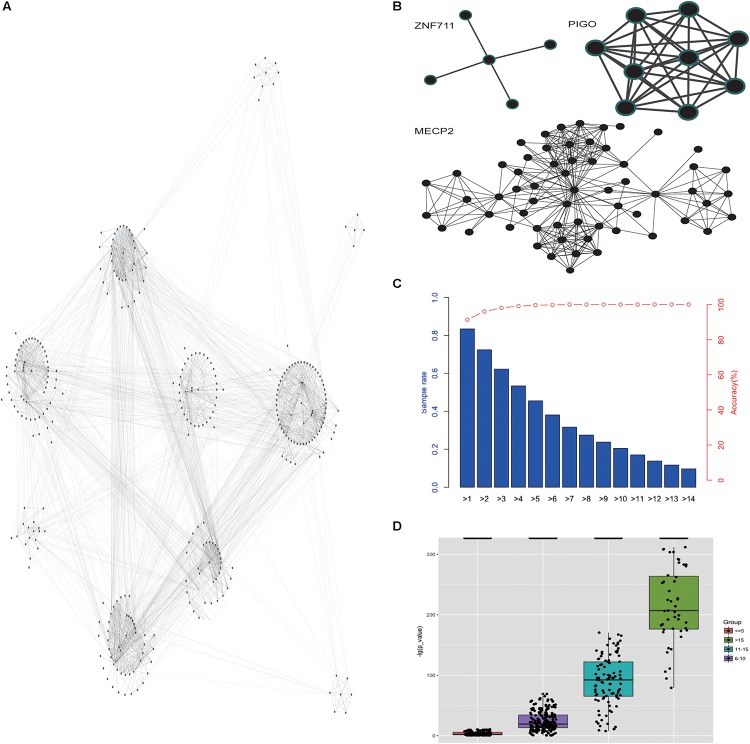
Gene-focused network for phenotype enrichment. **(A)** The network of phenotypes that were enriched in genes. **(B)** The three types of co-occurrence sub-networks. **(C)** The accuracy of the predicted pathogenic gene with phenotypes. **(D)** The *P*-value distribution of predicted pathogenic gene based on different number of given phenotypes.

Then we analyzed the network diagram of the phenotypic pairs for each gene, which revealed the gene-focused network (Figure 5A) and three typical sub-networks (Figure 5B). The first type of sub-network was radial, indicating that most of the phenotypes co-occurred with another one phenotype (like gene ZNF711). The pathogenic genes with the first type of sub-network may have a core phenotype, or an important phenotype that appeared more frequently, and it illustrated that there are strong association between the core phenotype and the biological function. The second type of sub-network was dense, and the phenotypes co-occurred with each other (like gene PIGO). The pathogenic genes with the second type of sub-network often result in a set of concurrent phenotypes. In this case, the prediction of pathogenic genes by phenotype may be more accurate. The third type of sub-network was the mixed state of the above two types (like gene MECP2). With the third type of sub-network of pathogenic genes, the mutations are usually more extensive, the phenotypes are complex, and one independent group phenotypes is often insufficient to reveal the pathogenic genes information. Our analysis showed that the co-occurrence network of each gene had its own characteristics, and the phenotypes in the co-occurrence network of each gene are different. And the co-occurrence networks of different genes had commonality in their structural similarity. Analysis of co-occurring networks further illustrated the phenotypic conservation relative to genes, despite the heterogeneity of phenotypes. Based on the above discoveries, we inferred that the pathogenic genes for patients could be achieved by analyzing specific phenotypes and phenotypic pairs. Our analyses indeed showed that the more the patients’ phenotypes, the more accurate the prediction of pathogenic genes (Figure 5C). Furthermore, given more phenotypes, the predicted pathogenic genes incline to have a more significant *P*-values (Figure 5D). These results showed that phenotypic analysis could reveal the convergences of ID phenotypes and be used for clinical pathogenic gene analysis.

### Pathogenic Gene Prediction

**FIGURE 6:**
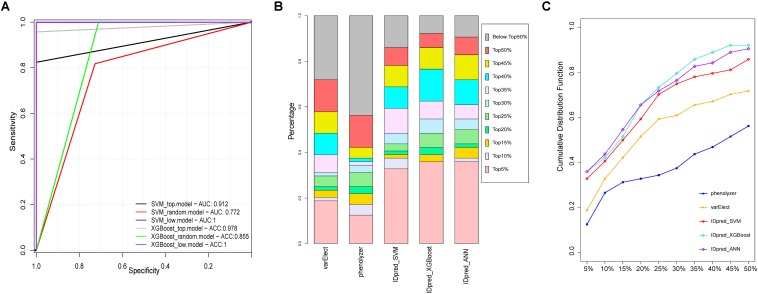
Performance comparison of pathological gene prediction between IDpred and other algorithms. **(A)** ROC curve derived from IDpred model based on 10 fold cross validation. **(B)** the percentage of predicted pathogenic gene derived from IDpred, phenolyzer, and varElect. **(C)** cumulative distribution of TopN rate base on the rank of the pathogenic gene derived from IDpred, phenolyzer, and varElect.

Support Vector Machine is one of the most widely used machine learning algorithms in computational biology. It was previously used for predicting virulent proteins in bacterial pathogens (Garg and Gupta, 2008), the clinical outcome from cancer patients (Yeoh et al., 2002) and gene interactions in genetic diseases (Upstill-Goddard et al., 2013). As shown in Supplementary Figure S1, developed SVM-based predictor, a 10-fold cross-validation was employed on the training datasets for model selection purpose (Figure 6A), and the final performance of the predictor was measured on the independent testing dataset (Ortiz-Gonzalez et al., 2018) compared with other ID pathogenic gene prediction models (Yang et al., 2015; Stelzer et al., 2016; Figures 6B,C). The receiver operating characteristic curve (sensitivity against 1-specificity) was used to measure the prediction performance under different decision thresholds, and the area under the curve (AUC) was calculated as the main performance evaluation metric. For calculating variable importance for prediction, 100 sets of independent training were performed using different random seed. The median of variable importance obtained in each training was used as a representative value (Supplementary Figure S2).

### Database and Tool for ID Research and Diagnosis

**FIGURE 7:**
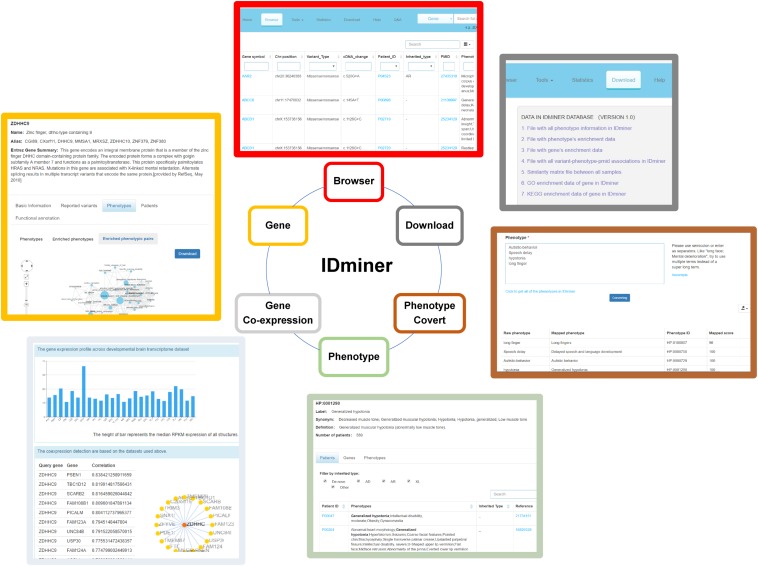
The illustration of functional modules of IDminer database. The six functional modules of IDminer: Brower, Genes, Gene co-expression, Phenotype, Phenotype convert, and Download.

In order to represent the ID data and the analysis tools for ID research and diagnosis, the IDminer system was designed. The database included a number of components, including a knowledge base for intellectual disabilities, specific phenotypes and phenotypic pairs for genes, co-occurrence networks, and analysis tools for converting phenotypes to standard phenotypes and exploring the expressions of interesting genes in the brain (Figure 7). IDminer was built on open sources software systems, such as MongoDB database, Express web development framework, Nginx web server, and Ubuntu operating system. Python and R were used for data collection processing and analysis. A user-friendly web interface was provided to help users search and analyze the data online at http://218.4.234.74:3100/IDminer/. The interface consists of seven parts: Home, Browser, Tools, Statistics, Download, Help, and Q&A. On the Home page, an introduction to the IDminer outlines a description statistic about all the data integrated into the database and the search module for gene and phenotype. There are two analysis tools for converting phenotypes and prioritizing candidate genes, respectively. Converting phenotypes is to help user mapping their clinical descriptions to our standard ID phenotypes, while co-expression analysis can be based on the brain gene expression data to study the expression profile of the interesting genes and its related genes. In the Document and Q&A pages, the guidelines for the database, and frequently asked questions and answers were showed. Furthermore, our database could be easily updated with the latest published information. For gene query, we provided basic gene information and linked it to multiple external databases, such as containing Ensemble, UniProtKB, GO, KEGG, and OMIM. Reported mutations, ID phenotypes, and patient information were also represented. Additionally, the gene’s phenotypic pairs were also interactively visualized. When users entered a phenotypic item in the input box, we listed its basic information such as HPO ID, synonyms and phenotype definitions, reported patients with this phenotype, reported causative genes causing the patients, and its co-occurrence network. For reported genes, in addition to displaying detailed mutation information of these genes, we also annotated the genes’ functions and performed PPI network analysis. Importantly, the query clinic feature could be enriched for some genes, and the genes were also listed. Finally, the top co-occurred phenotypic pairs ranked by their frequencies were shown as a network and the enriched genes for each pair were shown by clicking the edge.

### A Use Case for the IDpred

The case of a real patient with the pathogenic gene AAR2 and the standardized phenotypes [Microcephaly (HP:0000252), Cochlear malformation (HP:0008554), Hypoplasia of the corpus callosum (HP:0002079), Ventricular septal defect (HP:0001629), Global developmental delay (HP:0001263), Anteriorly placed anus (HP:0001545), Macule (HP:0012733), Patent foramen ovale (HP:0001655)] was selected based on the previous studies (Charng et al., 2016). The other input candidate genes were randomly selected from the gene list in our database. Then, the query genes list consisted of MXRA8, DMBX1, AAR2, CLIC2, PLA2G6, and phenotypes list consisted of all the standardized phenotypes of this patient (genes and phenotypes are separated by semicolons) were entered into the corresponding box on the page of the website. Then the selection of the models (for example, SVM) with the appropriate parameters should be submitted (Supplementary Figure S3A). The result page contains seven columns (GeneSymbol, PathogenicGeneRank, PathogenicScore, Pathogenicity, SimilarRank, SimilarScore, and Phenotypes) would be displayed. On the result table, PathogenicGeneRank is the rank of the input pathogenic genes compared to all deposited genes in our database, PathogenicScore is the score of the pathogenic genes, and Pathogenicity is defined as “Probably” (PathogenicScore > 0.5) or “Less likely” (PathogenicScore = 0.5). SimilarRank refers to the rank of similarity between gene and phenotypes, and SimilarScore refers to the calculated score of similarity between gene and phenotypes. Phenotypes listed the phenotypes related to the GeneSymbol. As shown in the result of this case, AAR2 was predicted as the pathogenic gene with the highest pathogenic score of 0.788 (Supplementary Figure S3B).

## Discussion

Our work manually extracted a large number of genes, clinic phenotypes and basic information of the patients from published ID literature. By integrating these data for comprehensive analysis, we have provided a holistic view of the current genetic research of ID and made the correlation of various clinic factors of ID patients, prompting researchers to further explore the mechanisms causing ID. The mutation spectrum delineated in our datasets provided essential information for molecular diagnosis in ID patients. Though most genes had its major mutation types, the spectrum showed that all mutation types were identified in ID cases. This combination of mutation types raises the need of using several clinical detection methods for ID diagnoses such as Array Comparative Genomic Hybridization (aCGH), target panel sequencing, whole exon sequencing, and even whole genome sequencing (De Ligt et al., 2013; Redin et al., 2014). Notably, because a small number set of genes accounted for most ID patients, targeted panel sequencing may be favorable than other methods in consideration of cost, time and the difficulty of the data analysis.

The phenotypes of ID patients were extremely diverse and heterogeneous. Unlike the previous study of phenotype-based clustering (Kochinke et al., 2016), we mapped the phenotypes of ID patients to HPO items and found the 2075 phenotypes in total 3803 patients. We confirmed not only mutations in different genes could lead to various phenotypes, but defects in a single gene had been implicated in different phenotypes. Interestingly, there was also considerable phenotypic heterogeneity even among individuals who have identical mutations in the disease gene. We speculated that, besides various genes, the heterogeneity of phenotypes could be affected by other factors, such as mutation types, genetic background, and environment. Though the phenotypes of ID patients were heterogeneous, the specific phenotypes for genes could be analyzed and used for prioritizing caused genes. A previous report suggests that, for tubulinopathies, each mutated gene has an associated predominant pattern of cortical dysgenesis (Bahi-Buisson et al., 2014). Additionally, the previous studies in ID found that convergent molecular pathways result in common phenotypes (Kochinke et al., 2016), allowing some phenotype-genotype correlation. However, the common phenotypes for each gene could be achieved until recently the applications of NGS, aCGH, target sequencing, WES, and WGS to ID patients, which lead to an increase of diagnosis. This larger sample size could raise the power of the statistical significance test. Then, for some genes, a large number of patients are sufficient to statistically to find the specific phenotypes, phenotypic pairs and co-occurrence networks for the genes. These features were extracted with enrichment in patients subgroup caused by each gene, confirming the phenotype-genotype correlations and the convergence of ID phenotypes among their extreme heterogeneities.

With the deepening of ID research and the increase of reported patients, it also requires the development of analytical tools for ID researchers to understand the data. Therefore, providing online friendly and easy-to-use analysis tools will also greatly assist in the research of the entire ID field. So, our website not only provides a knowledge base of ID but also aggregates tools commonly used in ID analysis. And more analysis tools for ID will be added in the future to promote ID research as much as possible.

Overall, our data and analysis showed the convergences of ID genes and phenotypes among their extreme heterogeneities. For genes, the convergence was characterized by the fact that a small percentage of genes could explain the majority of ID phenotypes. And for phenotypes, it was represented as genes’ specific phenotype and phenotypic pairs. Importantly, we provided analysis tools based on ID genes and phenotypes in hopes of establishing the standard ID gene and phenotype libraries and, in turn, aiding in clinical diagnosis. Overall, the findings and tools could contribute to the understanding of the genetic basis of ID disease and ultimately improve the diagnosis and treatment of the disease.

## Conclusion

Our analysis provided evidence to support, though the ID genes and phenotypes were extremely heterogeneous, the genetic bias and phenotypic convergence deserved our more attention, which may help to help us to quickly diagnose ID patients and further promote the studies of disease mechanisms. Moreover, our curated data, analysis, and developed tools were integrated to build a standard ID database IDminer, which could be accessed through http://218.4.234.74:3100/IDminer/. The database and interface are user-friendly for geneticists and clinicians, and a very wide range of ID researchers.

## Data Availability Statement

Publicly available datasets were analyzed in this study. This data can be found here: http://218.4.234.74:3100/IDminer/.

## Author Contributions

Z-CF, YW, L-NZ, and X-WM conceived the project, analyzed and interpreted the data, and wrote the manuscript. FY, X-LX, YY, XY, WP, W-QZ, J-YL, W-DZ, and X-LZ acquired data and performed bioinformatics analyses. T-JJ edited the manuscript. All authors read and approved the final manuscript.

## Conflict of Interest

The authors declare that the research was conducted in the absence of any commercial or financial relationships that could be construed as a potential conflict of interest.

## Footnotes

1 http://human-phenotype-ontology.github.io

2 http://geneontology.org/

3 http://www.genome.jp/kegg/

## Supplementary Material

FIGURE S1 The flowchart of pathogenic gene prediction model specification. The framework for training and test data generation, model construction, and validation.

FIGURE S2 Feature importance scores derived from IDpred. Feature importance is defined as the average gain of the feature in trees from XGBoost in IDpred_XGBoost.top, IDpred_XGBoost.random, and IDpred_XGBoost.low model.

FIGURE S3 Dpred interface and direct mode example. **(A)** The user enters three types of input: gene symbol list, phenotype expression and modeling type. **(B)** Output results presented in a tab with seven columns which were defined as GeneSymbol, PathogenicGeneRank, PathogenicScore, pathogenicity, SimilarRank, SimilarScore, and Phenotypes.

## References

[B1] Bahi-Buisson N. Poirier K. Fourniol F. Saillour Y. Valence S. Lebrun N. (2014). The wide spectrum of tubulinopathies: what are the key features for the diagnosis? *Brain* 137 1676–1700. 10.1093/brain/awu082 24860126

[B2] Charng W. L. Karaca E. Coban Akdemir Z. Gambin T. Atik M. M. Gu S. (2016). Exome sequencing in mostly consanguineous Arab families with neurologic disease provides a high potential molecular diagnosis rate. *BMC Med. Genomics* 9:42. 10.1186/s12920-016-0208-3 27435318 PMC4950750

[B3] De Ligt J. Willemsen M. H. Van Bon B. W. M. Kleefstra T. Yntema H. G. Kroes T. (2013). Diagnostic exome sequencing in persons with severe intellectual disability. *Obstet. Gynecol. Surv.* 68 191–193. 10.1097/01.ogx.0000428160.59063.a6 23033978

[B4] Ellison J. W. Rosenfeld J. A. Shaffer L. G. (2013). Genetic Basis of Intellectual Disability. *Annu. Rev. Med.* 64 441–450. 10.1146/annurev-med-042711-140053 23020879

[B5] Garg A. Gupta D. (2008). VirulentPred: a SVM based prediction method for virulent proteins in bacterial pathogens. *BMC Bioinformatics* 9:62. 10.1186/1471-2105-9-62 18226234 PMC2254373

[B6] Gécz J. Shoubridge C. Corbett M. (2009). The genetic landscape of intellectual disability arising from chromosome X. *Trends Genet.* 25 308–316. 10.1016/j.tig.2009.05.002 19556021

[B7] Gilissen C. Hehir-Kwa J. Y. Thung D. T. Van De Vorst M. Van Bon B. W. M. Willemsen M. H. (2014). Genome sequencing identifies major causes of severe intellectual disability. *Nature* 511 344–347. 10.1038/nature13394 24896178

[B8] Hoischen A. Krumm N. Eichler E. E. (2014). Prioritization of neurodevelopmental disease genes by discovery of new mutations. *Nat. Neurosci.* 17 764–772. 10.1038/nn.3703 24866042 PMC4077789

[B9] Kochinke K. Zweier C. Nijhof B. Fenckova M. Cizek P. Honti F. (2016). Systematic phenomics analysis deconvolutes genes mutated in intellectual disability into biologically coherent modules. *Am. J. Hum. Genet.* 98 149–164. 10.1016/j.ajhg.2015.11.024 26748517 PMC4716705

[B10] Lelieveld S. H. Reijnders M. R. F. Pfundt R. Yntema H. G. Kamsteeg E. J. De Vries P. (2016). Meta-analysis of 2,104 trios provides support for 10 new genes for intellectual disability. *Nat. Neurosci.* 19 1194–1196. 10.1038/nn.4352 27479843

[B11] Maulik P. K. Mascarenhas M. N. Mathers C. D. Dua T. Saxena S. (2011). Prevalence of intellectual disability: a meta-analysis of population-based studies. *Res. Dev. Disabil.* 32 419–436. 10.1016/j.ridd.2010.12.018 21236634

[B12] Mefford H. C. Batshaw M. L. Hoffman E. P. (2012). Genomics, intellectual disability, and autism. *N. Engl. J. Med.* 366 733–743. 10.1056/NEJMra1114194 22356326 PMC4107681

[B13] Ortiz-Gonzalez X. R. Tintos-Hernandez J. A. Keller K. Li X. Foley A. R. Bharucha-Goebel D. X. (2018). Homozygous boricua TBCK mutation causes neurodegeneration and aberrant autophagy. *Ann. Neurol.* 83 153–165. 10.1002/ana.25130 29283439 PMC5876123

[B14] Rauch A. Wieczorek D. Graf E. Wieland T. Endele S. Schwarzmayr T. (2012). Range of genetic mutations associated with severe non-syndromic sporadic intellectual disability: an exome sequencing study. *Lancet* 380 1674–1682. 10.1016/S0140-6736(12)61480-9 23020937

[B15] Redin C. Gérard B. Lauer J. Herenger Y. Muller J. Quartier A. (2014). Efficient strategy for the molecular diagnosis of intellectual disability using targeted high-throughput sequencing. *J. Med. Genet.* 51 724–736. 10.1136/jmedgenet-2014-102554 25167861 PMC4215287

[B16] Reichenberg A. Cederlöf M. McMillan A. Trzaskowski M. Kapara O. Fruchter E. (2016). Discontinuity in the genetic and environmental causes of the intellectual disability spectrum. *Proc. Natl. Acad. Sci. U.S.A.* 113 1098–1103. 10.1073/pnas.1508093112 26711998 PMC4743770

[B17] Stelzer G. Plaschkes I. Oz-Levi D. Alkelai A. Olender T. Zimmerman S. (2016). VarElect: the phenotype-based variation prioritizer of the GeneCards Suite. *BMC Genomics* 17(Suppl. 2):444. 10.1186/s12864-016-2722-2 27357693 PMC4928145

[B18] Upstill-Goddard R. Eccles D. Fliege J. Collins A. (2013). Machine learning approaches for the discovery of gene-gene interactions in disease data. *Brief. Bioinform* 14 251–260. 10.1093/bib/bbs024 22611119

[B19] Vissers L. E. L. M. Gilissen C. Veltman J. A. (2016). Genetic studies in intellectual disability and related disorders. *Nat. Rev. Genet.* 17 9–18. 10.1038/nrg3999 26503795

[B20] Yang H. Robinson P. N. Wang K. (2015). Phenolyzer: phenotype-based prioritization of candidate genes for human diseases. *Nat. Methods* 12 841–843. 10.1038/nmeth.3484 26192085 PMC4718403

[B21] Yeoh E.-J. Ross M. E. Shurtleff S. A. Williams W. K. Patel D. Mahfouz R. (2002). Classification, subtype discovery, and prediction of outcome in pediatric acute lymphoblastic leukemia by gene expression profiling. *Cancer Cell* 1 133–143. 10.1016/S1535-6108(02)00032-6 12086872

