# Supplementary figures and images for: Gene-Focused Networks Underlying Phenotypic Convergence in a Systematically Phenotyped Cohort With Heterogeneous Intellectual Disability
Source: Front Bioeng Biotechnol. 2020 Feb 7;8:45. doi: 10.3389/fbioe.2020.00045 (PMC7019181; doi:10.3389/fbioe.2020.00045)

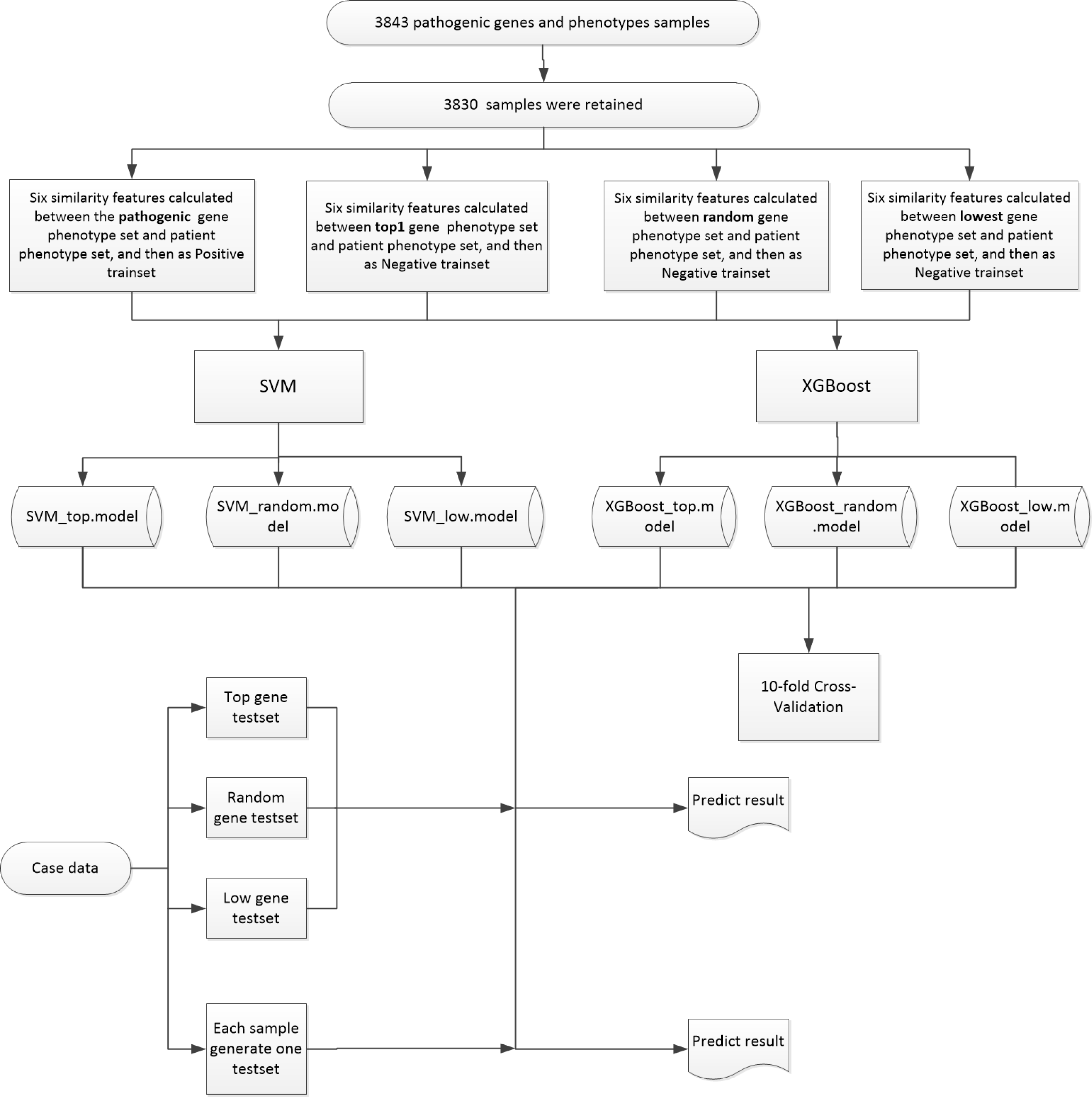

Supplement: FIGURE S1 — The flowchart of pathogenic gene prediction model specification. The framework for training and test data generation, model construction, and validation. [file Image_1.TIF]

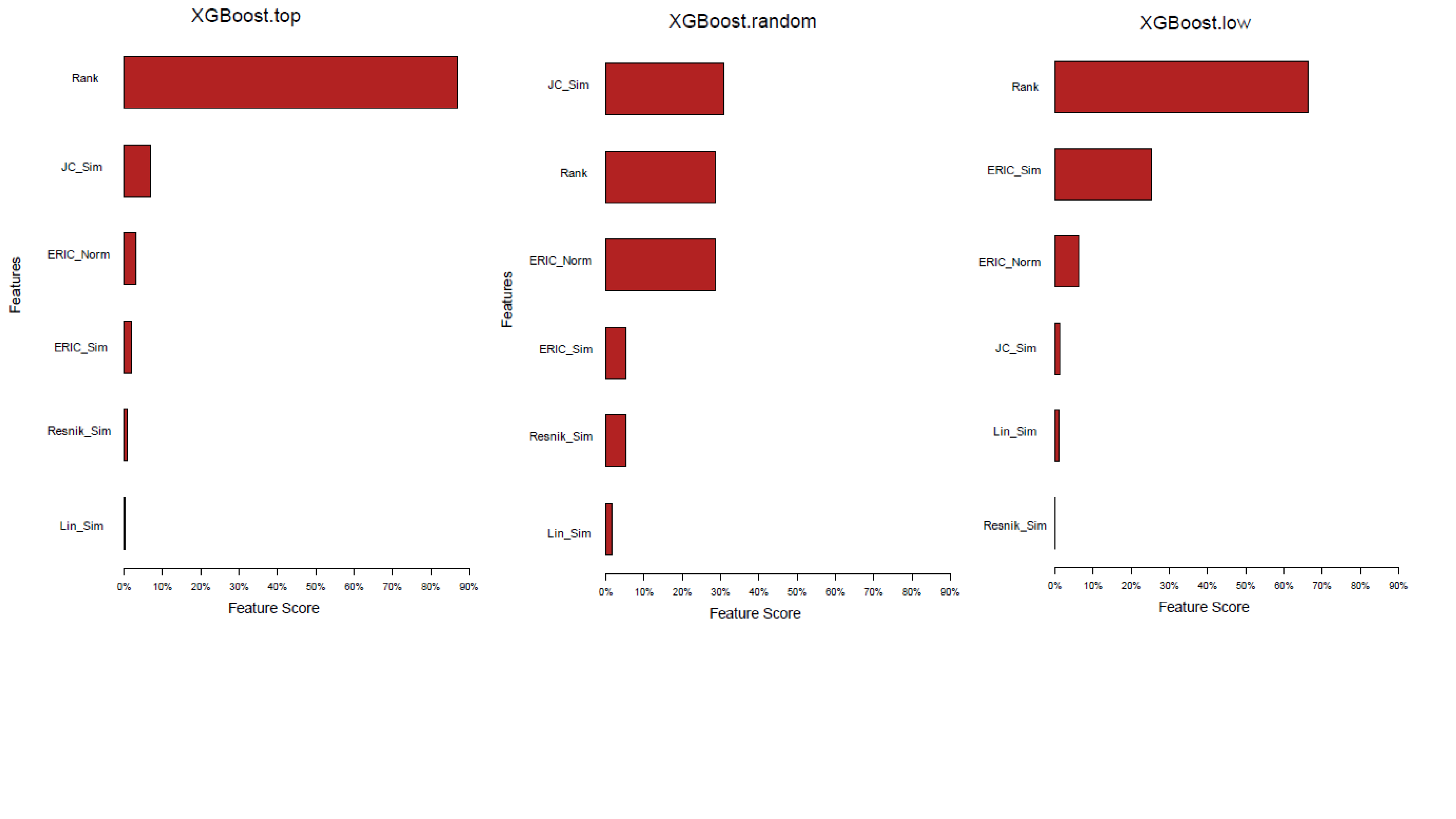

Supplement: FIGURE S2 — Feature importance scores derived from IDpred. Feature importance is defined as the average gain of the feature in trees from XGBoost in IDpred_XGBoost.top, IDpred_XGBoost.random, and IDpred_XGBoost.low model. [file Image_2.TIF]

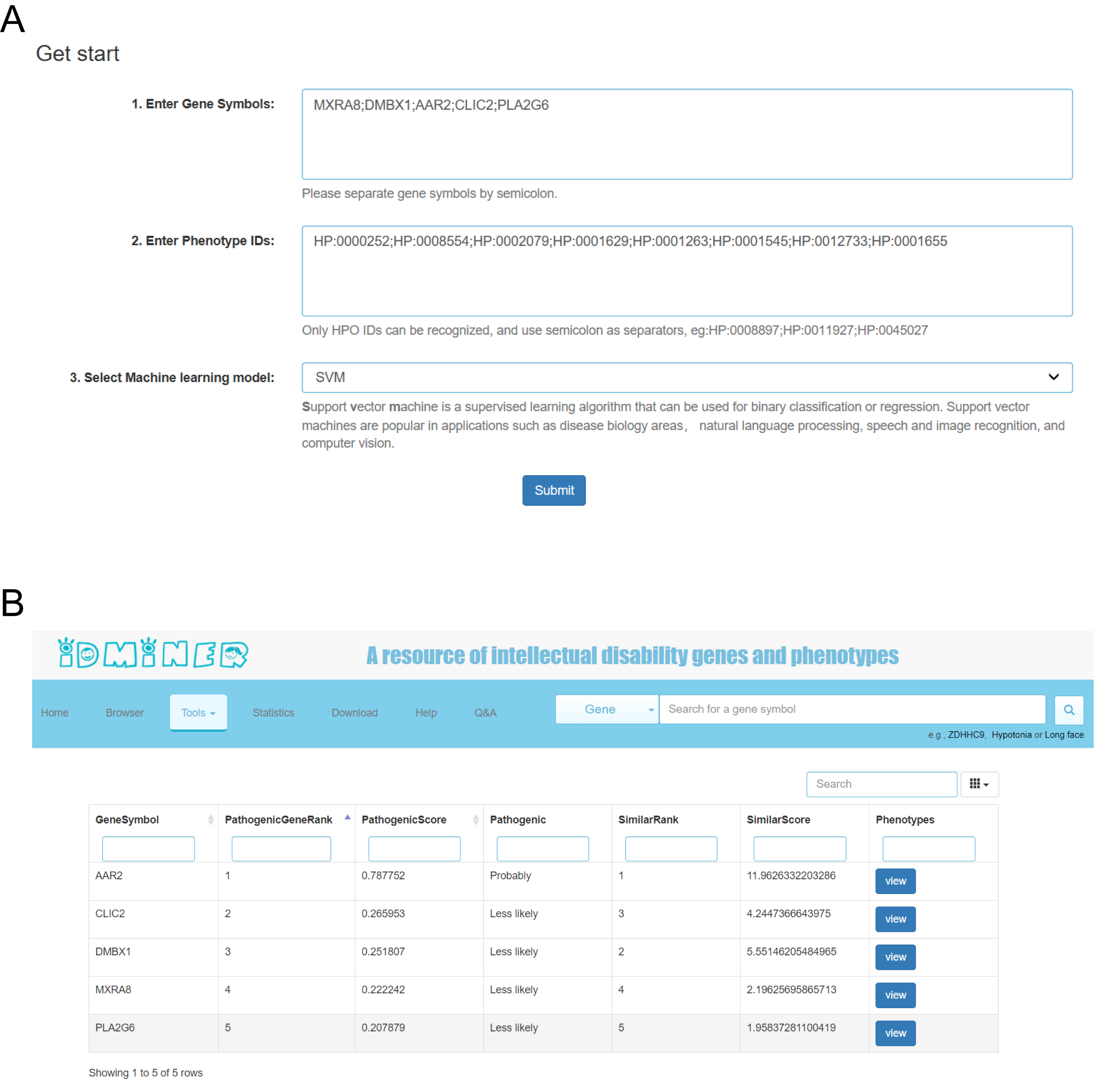

Supplement: FIGURE S3 — Dpred interface and direct mode example. (A) The user enters three types of input: gene symbol list, phenotype expression and modeling type. (B) Output results presented in a tab with seven columns which were defined as GeneSymbol, PathogenicGeneRank, PathogenicScore, pathogenicity, SimilarRank, SimilarScore, and Phenotypes. [file Image_3.TIF]
